# The logistic organ dysfunction system score predicts the prognosis of patients with alcoholic ketoacidosis

**DOI:** 10.1080/0886022X.2018.1491405

**Published:** 2019-02-11

**Authors:** Ha Nee Jang, Hee Jung Park, Hyun Seop Cho, Eunjin Bae, Tae Won Lee, Se-Ho Chang, Dong Jun Park

**Affiliations:** a Department of Internal Medicine, Gyeongsang National University Hospital, Jinju, South Korea;; b Department of Internal Medicine, Changwon Gyeongsang National University Hospital, Changwon, South Korea;; c Department of Internal Medicine, College of Medicine, Gyeongsang National University, Jinju, South Korea;; d Institute of Health Science, Gyeongsang National University, Jinju, South Korea

**Keywords:** Alcoholic ketoacidosis, LODS score, prognosis, mortality, acidosis

## Abstract

Alcoholic ketoacidosis (AKA) is occasionally associated with multiple complications leading to death. However, no study has yet evaluated prognostic factors in patients with AKA. It is known that the logistic organ dysfunction system (LODS) score is an objective and useful index to predict the prognosis. We used LODS score to predict prognosis of AKA. We retrospectively reviewed the medical records of 46 patients who were diagnosed as AKA in our hospital. The mean LODS score was 6.3. The probability of mortality based on the LODS score was 36.6%, and 16 patients (34.5%) did, in fact, die. The total LODS score and lactate dehydrogenase (LDH) were significantly higher in the non-survival group. Prothrombin activity, serum platelet number, and the serum albumin levels were significantly higher in the survival group. We found significant correlations between the LODS score and arterial pH, the albumin level, and the LDH concentration. Multivariate analysis showed that the serum albumin and LDH levels were independently associated with survival in AKA patients. AKA patients suffered high-level mortality and the LODS score was an accurate predictor of prognosis. Clinicians may use the LODS score to this end.

## Introduction

Alcoholic ketoacidosis (AKA) is usually characterized by metabolic acidosis, an increased anion gap, elevated serum ketone levels, and either a normal or low glucose concentration. A typical AKA patient has a history of chronic alcohol abuse, little or no food intake, and recent binge drinking followed by abrupt cessation of alcohol intake [1–6]. The physiological and mechanistic features of the condition are poorly known, but three factors may be in play. First, starvation may induce a decrease in insulin secretion and a rise in glucagon secretion. Second, the ratio of the level of the reduced form of nicotinamide adenine dinucleotide (NADH) to the oxidized form (nicotinamide adenine dinucleotide (NAD^+^)) may become elevated during alcohol metabolism. Finally, the extracellular fluid volume may be depleted by vomiting and low fluid intake [5–8]. The principal symptoms are nausea, vomiting, and/or abdominal pain [5,6]. Although the symptoms improve upon conservative management (infusion of large amounts of normal saline and glucose solution), AKA is occasionally associated with multiple complications [9,10].

Two reports found that 5–10% of chronic alcoholics died unexplained deaths (thus, all possible etiologies had been thoroughly investigated) [11,12]. Medico-legal autopsies revealed that AKA was responsible for 7% of deaths attributable to chronic alcoholism [13]; forensic pathologists sometimes report unexpected deaths [14,15]. AKA is associated with multiple complications including acute pancreatitis, acute renal failure (ARF) (caused by rhabdomyolysis), Wernicke’s encephalopathy, and sepsis accompanied by acute respiratory distress syndrome (ARDS) [9,10,16]. Several clinical reports have described life-threatening metabolic disorders in, or sudden death of, chronic alcoholics, associated with either ketoacidosis or lactic acidosis [17–22]. Also, sudden cardiac arrest has been associated with AKA [10].

The logistic organ dysfunction system (LODS) score is an objective and useful index. The LODS assesses six systems in terms of the extent of dysfunction on the first day of an intensive care unit (ICU) stay; the systems evaluated are the neurologic, cardiovascular, renal, pulmonary, hematologic, and hepatic systems. Physiological variables can predict mortality; the LODS score is converted to a probability of death [23–25]. Recently, we found that our AKA patients had poor prognoses and hypothesized that the LODS score might yield prognostic information. We therefore conducted this retrospective study.

## Materials and methods

We retrospectively reviewed the medical records of 46 patients diagnosed with AKA from January 2009 to December 2013 in Gyeongsang National University Hospital. The study protocol was approved by the Institutional Review Board at Gyeongsang National University Hospital (IRB No: 2014–05-026).

AKA was diagnosed (a) by a characteristic history of chronic alcohol abuse plus a recent binge, followed by binge termination, severe nausea, vomiting, and abdominal pain; (b) typical clinical findings including tachycardia, hypotension, an increased respiratory rate, and abdominal tenderness; (c) biochemical features including an elevated anion gap, a normal or low blood glucose level, and ketone bodies in the plasma (as revealed by the nitroprusside reaction (Ketostix); the assay is most sensitive to acetoacetate, less so to acetone, and not at all to beta-hydroxybutyrate (BOHB)); and/or (d) an elevated plasma BOHB or acetoacetate level. Patients with diabetic or starvation-induced ketoacidosis, or lactic acidosis attributable to other causes, were excluded.

The values for LODS (six systems) and various laboratory values were described on the basis of initial values sampled on visiting the emergency room and all patients underwent above tests. This score accurately measures the severity of system dysfunctions (neurologic, cardiovascular, renal, pulmonary, hematologic, and hepatic). The total score varies from 0 (all normal) to 22 (all systems severely compromised), and a predicted mortality score is subsequently calculated automatically. Acute kidney injury (AKI) was defined using the risk, injury, failure, loss of kidney function, and end-stage kidney disease (RIFLE) criteria. As 6- and 12-h urine volumes were lacking, we used the serum creatinine level and the glomerular filtration rate (GFR) to determine the RIFLE classification. GFR was calculated using the simplified “modification of diet in renal disease” formula: GFR = 186 × [serum creatinine level] – 1.154 × [age in years] – .023 × [.742 if female] × [1.210 if black]. Data obtained in the first 24 h after admission were used; these were arterial blood gas information (pH, bicarbonate level, PaO_2_, and PaCO_2_); blood urea nitrogen (BUN) level; serum creatinine level; electrolyte (sodium, chloride, potassium, calcium, and phosphorus) concentrations; the serum anion gap; the arterial lactate level; leukocyte number; hemoglobin level; platelet counts; C-reactive protein (CRP), albumin, lactate dehydrogenase (LDH), amylase, and lipase levels; and prothrombin (PT) activity expressed as a percentage of the normal level. Acute pancreatitis was diagnosed when amylase and/or lipase levels were at least threefold greater than the normal values.

All values are expressed as means ± standard deviations (SDs). Pearson’s chi-squared test was used to explore the significance of qualitative differences. The parametric Student’s t-test was employed to compare the means of samples similar in terms of variance. Bivariate analysis was used to determine correlations between continuous variables. Multivariate logistic regression analysis was performed to identify which risk factors for death, identified upon simple regression, remained significant. All statistical analysis was performed with the aid of SPSS for Windows version 18.0 (SPSS, Chicago, IL, USA). A *p* value <.05 was taken to indicate statistical significance.

## Results

### Baseline characteristics

We included a total of 46 patients and their clinical and biological characteristics are shown in Table 1. Of all patients, 87% were males. At admission, 50% of patients were in shock and required vasoactive support; 39% needed mechanical ventilation, and 85% had AKI diagnosed using the RIFLE criteria. Ten patients had acute pancreatitis. The mean arterial pH was 6.97. Renal replacement therapy was required by 14 patients.

**Table 1.** Main clinical and biological characteristics of patients at admission.

Characteristics	*n* = 46
Age (yr)	49.67 ± 9.78
Male/female, *n* (%)	40/6 (87.0)
Initial systolic blood pressure (mm/Hg)	109.09 ± 33.85
Inotropic support at admission, *n* (%)	23 (50)
Need for mechanical ventilation, *n* (%)	18 (39.1)
Renal replacement therapy, *n* (%)	14 (30.4)
AKI, *n* (%)	39 (84.8)
Risk	9 (23.1)
Injury	11 (28.2)
Failure	19 (48.7)
Acute pancreatitis, *n* (%)	10 (21.7)
Total LODS score	6.33 ± 4.47
Probability of mortality based on the LODS score (%)	36.62 ± 31.17
Arterial pH	6.97 ± .24
Bicarbonate (mmol/L)	4.94 ± 3.57
BUN (mmol/L)	29.67 ± 19.61
Serum creatinine (mmol/L)	2.27 ± 1.55
Plasma lactate (mmol/L)	14.35 ± 8.53
Serum anion gap (AG) (mmol/L)	39.69 ± 10.09
Corrected AG (mmol/L)	40.54 ± 9.73
Prothrombin activity (%)	58.27 ± 21.30
Total bilirubin	1.86 ± 1.54
White blood cell (x10^3^/mm^3^)	12.84 ± 12.16
Hemoglobin (g/dL)	12.65 ± 2.81
Platelet (x10^3^/mm^3^)	178.74 ± 105.12
Albumin (g/dL)	3.63 ± .92
Amylase (U/L)	247.64 ± 438.04
Lipase (U/L)	109.86 ± 149.31
LDH (IU/L)	779.11 ± 1076.45
CRP (mg/L)	20.50 ± 42.38

LODS: Logistic organ dysfunction system.

### Prognostic factors

At admission, the mean probability of mortality based on the LODS score was 36%. In fact, 16 of the 46 patients (35%) died in hospital (Tables 1 and 2). Data from the univariate comparison of survivors and non-survivors are shown in Table 2. Inotropic support, mechanical ventilation, and renal replacement therapy were more frequently required by non-survivors (*p* = .029, .005, and .048, respectively). The frequencies of AKI (diagnosed using the RIFLE criteria) and acute pancreatitis did not differ between the two groups. Both the total LODS score and the probability of mortality yielded by the score were higher in non-survivors (*p* = .011 and .009, respectively). None of arterial pH, plasma lactate level, the plasma crude or corrected anion gap, the white blood cell count, or the hemoglobin or CRP level differed between the groups. Prothrombin activity, serum platelet count, and the albumin level were significantly lower (*p* = .012, .004, and .017, respectively), and the LDH level significantly higher, in non-survivors (*p* = .018). The relative risks of death yielded by univariate component analysis of arterial pH < 7.0, a total LODS score >6, a serum albumin level <3.0 g/dL, and an LDH level >800 IU/L are shown in Table 3. The total LODS score and the serum albumin LDH level were significant predictors of prognosis. Multivariate analysis was used to define factors among the above variables independently associated with mortality (Table 3). The serum albumin and LDH levels were significant predictors of prognosis (odds ratio (OR): 26.37 [1.6–436.8], *p* = .022; and OR: 41.17 [2.55–665.49], *p* = .009, respectively), whereas arterial pH and the LODS score were not.

**Table 2.** Comparison between survivors and non-survivors.

Characteristics	Survivors (30)	Non-survivors (16)	*p* Value
Age (yr)	49.23 ± 10.48	50.50 ± 8.56	.680
Male/female, *n* (%)	25/5 (83.3)	15/1 (93.8)	.318
Initial systolic blood pressure (mm/Hg)	108.57 ± 33.67	110.06 ± 35.28	.888
Inotropic support at admission, *n* (%)	11 (36.7)	12 (75.0)	.029
Need for mechanical ventilation, *n* (%)	7 (24.1)	11 (68.8)	.005
Need for renal replacement therapy, *n* (%)	6 (20.0)	8 (50.0)	.048
AKI, *n* (%)	24 (80.0)	15 (93.8)	.464
Risk	4 (16.7)	5 (33.3)	
Injury	6 (25.0)	5 (33.3)	
Failure	14 (58.3)	5 (33.3)	
Acute pancreatitis (%)	6 (20.7)	4 (25.0)	
Total LODS score	5.13 ± 4.04	8.56 ± 4.49	.011
Probability of mortality based on the LODS score (%)	28.08 ± 27.20	52.61 ± 32.65	.009
Arterial pH	7.01 ± 0.22	6.89 ± 0.27	.115
Bicarbonate (mmol/L)	4.73 ± 3.12	5.31 ± 4.39	.606
BUN (mmol/L)	30.82 ± 21.48	27.51 ± 15.92	.591
Ceatinine (mmol/L)	2.32 ± 1.73	2.17 ± 1.19	.749
Lactate (mmol/L)	13.05 ± 9.21	16.12 ± 7.46	.315
Serum anion gap (AG) (mmol/L)	40.46 ± 10.29	35.49 ± 9.14	.112
Corrected AG (mmol/L)	40.85 ± 9.84	37.51 ± 9.44	.273
Prothrombin activity (%)	64.10 ± 19.94	47.69 ± 20.10	.012
Total bilirubin	1.71 ± 1.19	2.06 ± 0.51	.371
White blood cell (x10^3^/mm^3^)	9.70 ± 9.33	16.48 ± 16.13	.077
Hemoglobin (g/dL)	12.96 ± 3.05	12.06 ± 2.23	.308
Platelet (×10^3^/mm^3^)	210.20 ± 102.26	119.75 ± 85.10	.004
Albumin (g/dL)	3.86 ± 0.83	3.19 ± 0.93	.017
Amylase (U/L)	157.56 ± 183.68	409.80 ± 674.88	.073
Lipase (U/L)	90.89 ± 120.47	144.00 ± 190.77	.275
LDH (IU/L)	501.69 ± 598.44	1520.52 ± 380.13	.018
CRP (mg/L)	18.26 ± 38.69	24.66 ± 49.81	.654

LODS: Logistic organ dysfunction system.

**Table 3.** Univariate and multivariate analyses for risk factors for death associated with AKA.

Characteristics	Univariate analysis			Multivariate analysis		
	*p* value	OR	95% CI	*p* value	OR	95% CI
pH <7.0	.129	2.877	.800–10.35	.058	13.955	.913–213.26
Total LODS score >6	.035	4.250	1.090–16.62	.545	1.819	.262–12.632
Albumin <3.0g/dL	.032	5.056	1.190–21.41	.022	26.373	1.592–436.78
LDH >800 IU/L	.000	28.000	3.300–258.4	.009	41.169	2.547–665.485

OR: Odds ratio, LDH: lactate dehydrogenase, LODS: logistic organ dysfunction score, CI: confidence interval.

### Correlations between the LODS score and biological variables

**Figure 1.**
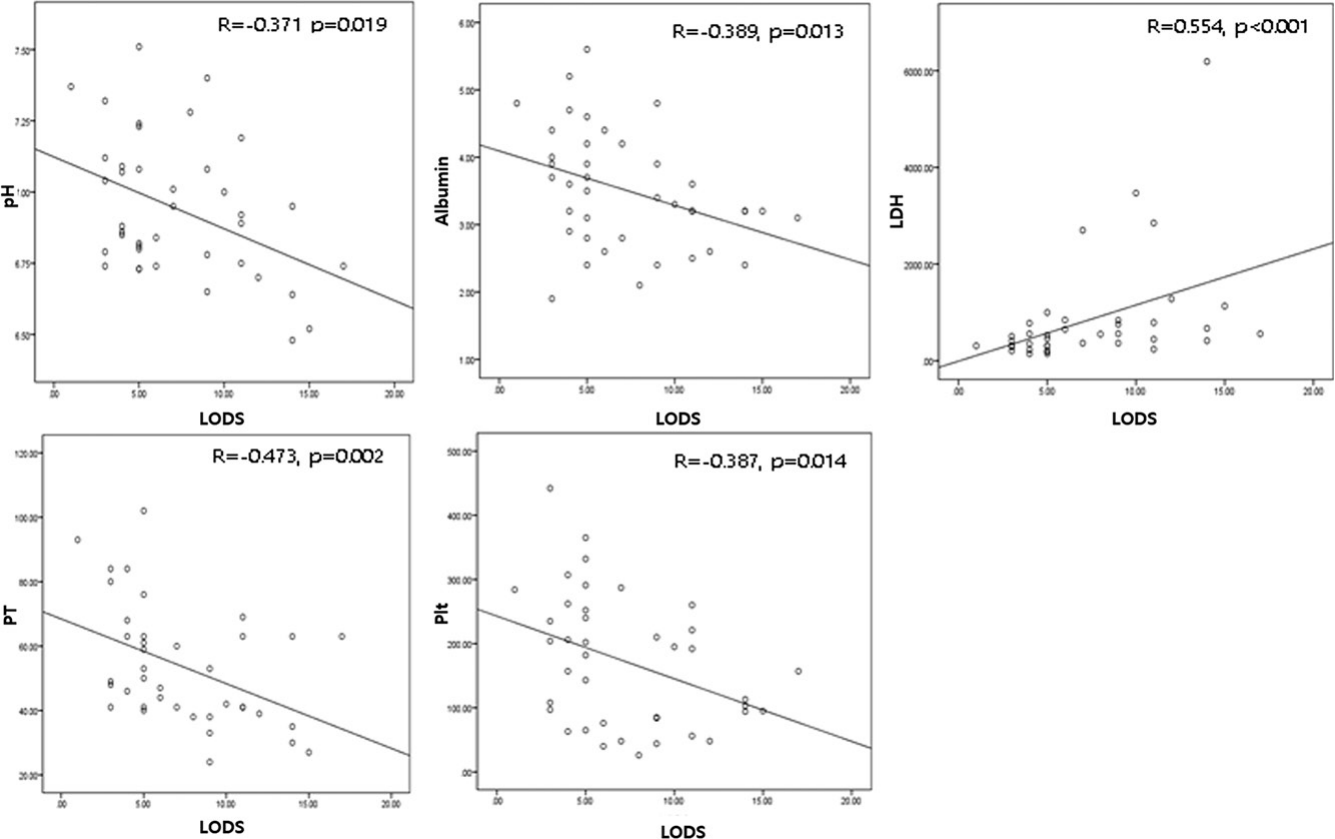
Correlation between LODS score and arterial pH, serum albumin level, LDH, prothrombin activity, and platelet number.

**Figure 2.**
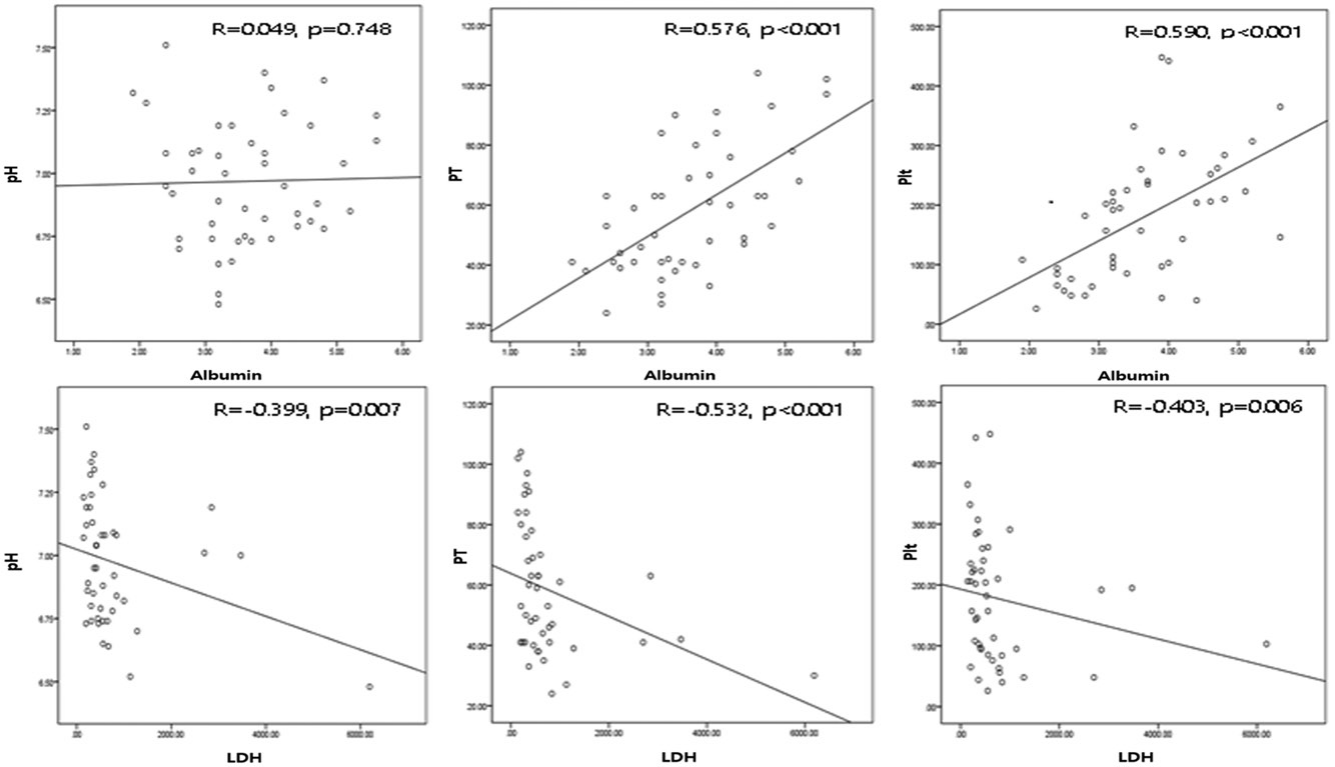
Correlation between serum albumin and LDH level and arterial pH, prothrombin activity, and platelet number.

We found significant correlations between the LODS score and arterial pH (*r* = –.371, *p* = .019), serum albumin level (*r* = –.389, *p* = .013), serum LDH level (*r* = .554, *p* < .001), prothrombin activity (*r* = –.473, *p* = .002), and platelet number (*r* = –.387, *p* = .014) (Figure 1). The serum albumin level also correlated positively with prothrombin activity and platelet number (*r* = .576, *p* < .001 and *r* = .590, *p* < .001, respectively), but not arterial pH (*r* = .049, *p* = .748). Serum LDH level significantly correlated with all of arterial pH, prothrombin activity, and platelet number (*r* = –.399, *p* = .007, *r* = –.532, *p* < .001, and *r* = –.403, *p* = .006, respectively) (Figure 2).

## Discussion

We found that AKA was associated with high mortality, and that prognostic factors included the LODS score, prothrombin activity, platelet number, and albumin and LDH levels (especially the latter two factors).

The LODS score calculated on admission accurately predicts both early and late ICU mortality [24–27]. The score also accurately assesses disease severity and organ dysfunction in critically ill patients such as those with severe sepsis and/or ARDS [28–31]. The prognosis of patients with metformin-associated lactic acidosis was reflected by their LODS scores, which precisely measured multiple organ dysfunctions [32]. Thus, we initially used this scoring system to predict the prognosis of AKA patients; we had earlier noted that some patients had poor prognoses. Upon single-variable analysis, the LODS score was significantly associated with prognosis. In particular, the probability of mortality yielded by the LODS score was very close to the actual mortality (36% vs. 34.8%). Thus, the LODS score can be used to predict AKA severity.

Although AKA patients usually have high plasma levels of free fatty acids, severe acidemia is uncommon because concurrent conditions including volume depletion, alcohol withdrawal, pain, and severe liver disease create mixed acid/base disturbances [4,5]. We found that acidemia (as reflected by the plasma bicarbonate level and pH) was generally absent; the bicarbonate level did not differ between survivors and non-survivors. A previous report found that sudden unexplained deaths occurred in 10% of chronic alcoholics. Thorough necropsies, standard toxicology screens, and scene investigations were not informative [19]. The cited authors proposed that severe electrolyte derangement (involving potassium and/or magnesium levels) might explain the sudden deaths. We found no difference in sodium or potassium levels between survivors and non-survivors.

The plasma BOHB level of AKA patients correlates with the frequency of unexplained death [18,19]. Kadis et al. [18] found that BOHB levels were significantly elevated in alcoholic patients who died such deaths, as compared to those whose deaths could, in fact, be explained. Denmark also considered that plasma BOHB was the principal cause of unexplained death in chronic alcoholics and proposed that alcoholic ketosis may have triggered fatal hypoglycemia [19]. In our present study, the plasma BOHB level did not significantly differ between survivors and non-survivors, being in fact slightly higher in survivors (data not shown). However, we cannot state that the BOHB level was not a good indicator of prognosis because this was measured in only six patients. We did not encounter any hypoglycemia. Thomsen et al. suggested that acidosis per se could derange vital metabolic functions and cause death [13]. The cited authors described unexpected deaths in AKA patients as “ketoalcoholic deaths” and subsequently reported that 7% of all sudden deaths in a prospective series of alcoholics were of this nature [17]. Pounder et al. [22] also proposed that AKA accompanied by high levels of ketone bodies might explain 10% of sudden hitherto unexplained deaths in alcoholics. We completely agree with the view of the cited authors that AKA per se causes sudden death via either a direct toxic effect or another unknown mechanism.

The LDH level was the best predictor of AKA patient death. A high LDH level usually indicates that cell turnover is elevated; this is a feature of various injuries. LDH levels are high in patients with cancer, hemolysis, tissue breakdown, liver disease, and pancreatitis. Although we do not know why the LDH level correlated positively with mortality, underlying chronic liver disease and/or acute liver injury, heart disease, rhabdomyolysis, and/or pancreatitis may have elevated the LDH level. Albumin level and the prothrombin component (a component of the LODS score) also correlate with the severity of chronic liver disease. These indices also correlated positively with AKA prognosis. The LODS score exhibited significant predictive power upon single-variable, but not multivariable, analysis. We consider that other parameters, such as the LDH level, predict prognosis very accurately, thus attenuating the power of the LODS score upon multivariable analysis.

Our study had certain limitations attributable to its retrospective nature. First, we could not thoroughly evaluate the causes of death because those who died did so soon after admission. Most patients died within 3 days; neither the causes of death nor underlying diseases were subjected to thorough work-up, especially in patients who visited the hospital on public holidays. Second, acetoacetate and BOHB levels were available for only a few patients, compromising statistical power. Third, AKA patient numbers were low, and mortality high, again attenuating the statistical power. The high mortality is explained by the fact that our hospital is a regional facility; most critical patients are transferred to us from other hospitals but mild cases are not. Therefore, our patients may not have exhibited typical AKA characteristics.

## Conclusions

AKA per se is commonly associated with a poor prognosis, as are the several complications that may accompany AKA; the LODS score accurately reflects this. Therefore, LODS score might be helpful to predict the prognosis of AKA patients.

## Ethical approval

The study protocol was approved by the Institutional Review Board at Gyeongsang National University Hospital (IRB No: 2014–05-026).
